# A qualitative study on professionals’ attitudes and views towards the introduction of patient reported measures into public maternity care pathway

**DOI:** 10.1186/s12913-021-06658-z

**Published:** 2021-07-03

**Authors:** An Chen, Kirsi Väyrynen, Riikka-Leena Leskelä, Seppo Heinonen, Paul Lillrank, Aydin Tekay, Paulus Torkki

**Affiliations:** 1 Department of Industrial Engineering and Management, Institute of Healthcare Engineering, Management and Architecture (HEMA), Aalto University, Maarintie 8, P.O. Box 15500, FI-00076 AALTO, 02150 Espoo, Finland; 2 Nordic Healthcare Group Oy, Vattuniemenranta 2, 00210 Helsinki, Finland; 3 Department of Obstetrics and Gynaecology, Central Finland Central Hospital, Keskussairaalantie 19, 40620 Jyväskylä, Finland; 4 Department of Obstetrics and Gynaecology, Helsinki University Hospital and University of Helsinki, Haartmaninkatu 2, 00290 Helsinki, Finland; 5 Department of Public Health, Faculty of Medicine, Helsinki University, Biomedicum 1, 00290 Helsinki, Finland

**Keywords:** Patient-reported measures (PRMs), Patient-reported outcome measures (PROMs), Patient-reported experience measures (PREMs), Health outcomes, Maternity care, Pregnancy, Childbirth, Implementation, Qualitative research

## Abstract

**Background:**

The importance and potential benefits of introducing patient reported measures (PRMs) into health care service have been widely acknowledged, yet the experience regarding their implementation into practice is limited. There is a considerable paucity of research in adopting PRMs in maternity care routine. This study, which utilizes the PRMs included in Pregnancy and Childbirth (PCB) outcome set developed by International Consortium for Health Outcomes Measurement (ICHOM) as sample measures, aims to elicit Finnish professionals’ views on PRMs and to explore the applicability of PRMs in Finnish public maternity care.

**Methods:**

This qualitative study, applying semi-structured interviews, described the local professionals’ views towards the application of PRMs in Finnish public maternity care. Professionals were asked to assess the PRMs defined in ICHOM PCB set and provide their expectations and concerns on the implementation of PRMs in Finnish public maternity service.

**Results:**

Twenty professionals participated in the interviews. Participants agreed on the importance and relevance of the PRMs questions included in ICHOM PCB set for delivering and developing maternity care in Finland. However, they criticized the number and length of questions as well as the recommended time points of data collection. In addition, for a successful implementation, various steps like developing suitable questions, redesigning service pathway and protocols, and motivating women to respond to PRMs questions were considered to be important. Also, some potential obstacles, difficulties and risks associated with the implementation were underlined.

**Conclusion:**

This study indicates that the implementation of PRMs into Finnish public maternity service is possible, highly relevant and important. However, the adoption of PRMs into routine practice may be challenging and will require a series of efforts. This study shows viewpoints from Finnish professionals who have not participated in developing the ICHOM PCB standard set and provides important insights on the development and implementation of PRMs.

**Supplementary Information:**

The online version contains supplementary material available at 10.1186/s12913-021-06658-z.

## Introduction

Patient-reported measures (PRMs) gather information about the outcomes and the experiences of health services as perceived and described by patients. PRMs help service providers understand what matters to patients, and they have become accepted as a core dimension of healthcare quality [1, 2]. Increasing the use of these measures and incorporating them into existing measurement dashboards are considered as effective strategies to achieve patient-centered care (PCC) and value-based health care (VBHC), a widely accepted healthcare delivery model introduced by Michael Porter [1, 3–8]. In PRMs, patient-reported outcome measures (PROMs) capture patients’ perceptions of their health status and help evaluate the result of a clinical intervention, while patient-reported experience measures (PREMs) collect information on patients’ personal experience of the healthcare services they have received [6]. Simple and occasional surveys on patient perception and satisfaction without standardized, systematic and comprehensive patient reported measures are inadequate for providers to capture the true feelings of patients or the gaps in the delivery of the services. By implementing PRMs to complement clinical outcome measures, healthcare professionals and providers can detect unrecognized health problems, effectively monitor and evaluate the patient’s health status holistically and longitudinally whether health care services have an effect on patients, assess whether the clinical outcomes are relevant for the patient, and communicate better with patients [9–11]. With PRMs, the patient will be able to make informed decisions and assume some responsibility for the management of their health conditions [12]. For healthcare administrators, PRMs provide an opportunity to identify care gaps and understand vulnerable groups in healthcare [1].

Although the importance and potential benefits of integrating PRMs into healthcare outcome measurement and evaluation have been widely acknowledged and use of PRMs has rapidly grown in recent decades, applicable knowledge and experience of structurally and routinely collecting and using PRMs data in clinical practices are still limited [2]. Most of the PRMs-implementation studies published in the past decade focus on particular medical areas, such as oncology, palliative care, mental health, and chronic diseases [13, 14], or on specific medical stages or clinical episodes, such as elective surgery [5]. Research-based evidence of acceptability, feasibility, and impact of implementing PRMs across the pathway of pregnancy and childbirth is still insufficient, and context-sensitive strategies and practices of successfully integrating PRMs into maternity care routine are also lacking [13].

Pregnancy and childbirth are life-changing events for women and families, consisting of physical, psychological and emotional changes as well as personal experiences during the whole process [15] that PRMs could be applied to capture. A research team from Italy advocated a “patient pathway” approach for developing a healthcare performance evaluation system to serve the regional and local health authorities’ managerial needs, and presented an example of systematically and structurally measuring, evaluating and reporting the performance of the whole maternity care pathway at regional level with the multi-dimensional indicators including patient reported satisfaction and experiences at different phases of care pathway [16–18]. The benefits of the routine collection of PREMs across the maternity care pathway, e.g. allowing healthcare authorities and providers to be aware of the quality of care along the whole care pathway as perceived by the patients, which helps identify gaps and insufficiencies in service provision, have been suggested in several studies by the Italian team [19–23]. However, more evidence-based, transferrable and practical knowledge about the implementation of this kind of system is needed for practitioners and researchers from other countries or institutions to integrate PRMs into local maternity care pathway. Interesting questions are, how this kind of pathway-based measurement system could be successfully developed, implemented and rolled out, how it could be used in clinical routine to guide frontline professionals’ decisions and practices to consider patients’ preferences and meet their needs, and whether this system would be adaptable and applicable in different contexts. The Italian studies did not include standard PROMs. A variety of available methods, tools and instruments could potentially be helpful in developing PREMs and PROMs to address general or specific issues across the pathway of pregnancy and childbirth [24–29]. A comprehensive and international standardized set of PROMs and PREMs for maternity care has been developed by the International Consortium on Health Outcome Measurement (ICHOM) [30], a not-for-profit organization that aims to facilitate the adoption of value-based health care worldwide. ICHOM published an outcomes and quality measurement set for pregnancy and childbirth (PCB set) in 2018. This set covers the full cycle of care with five critical data-collection time points and contains PROMs (e.g. health related quality of life, pain with intercourse, and postpartum depression) and PREMs (e.g. satisfaction with care, confidence in healthcare providers and confidence in healthcare providers) (Additional file 1 displays PROMs and PREMs and other patient reported items defined in ICHOM PCB set) [31]. Although ICHOM has provided a reference guide with hands-on materials [32] to facilitate the process of implementing this standard set into routine practice, a complete pre-implementation investigation of local acceptability and applicability is required. Until recently, we are aware of only few studies formally describing and assessing the local adoption of the PRMs defined in ICHOM PCB outcome set, with two studies from Netherlands and one from Kenya [13, 33, 34]. Implementation research on maternity-related PRMs is quite limited. The lack of an extensive knowledge base and structural guidance seems to hinder the routine collection and use of PRMs in maternity care pathway, where PRMs could bring considerable value. The implementation of PRMs are affected by a wide range of factors, e.g. service contexts, institutional characteristics, health conditions of populations, and local culture [14, 35]. More context-based knowledge and transferrable insights of the implementation of maternity-related PRMs should be developed. A complete pre-implementation investigation of local acceptability and applicability is indispensable.

Finnish public maternity care system has obtained international acclaims for its lowest maternal mortality rate as well as for the equity and equality in distributing the services among women and their families. However, as the bar for quality raises, and patient-centered and value-based care become a megatrend, the evaluation of the quality of care requires new insights. Until now, Finland, as many other countries, has not established well-structured and proper measures that can systematically reflect women’s and their families’ views and experiences on the process and outcomes of care they receive. The implementation of PRMs into Finnish public maternity care could solve these problems. Introducing PRMs for developing VBHC and PCC has been in healthcare administration’s agenda.

This study, using PROMs and PREMs defined in ICHOM PCB set as sample measures and with the context of Finnish public maternity care, aims to explore the acceptability and feasibility of implementing PRMs in maternity care pathway. We expect this study to provide useful evidence to policy-makers and service providers involved in planning the implementation of maternity-related PRMs, and add context-based insights to the knowledge base of PRMs implementation. There is a wide consensus that frontline healthcare professionals are the key stakeholders playing an integral role in the implementation of PRMs, especially in the routine use of PRMs data, and investigating their attitudes and views of toward PRMs is essential prior to pilot implementation [14, 36]. The multidisciplinary system and professional specializations of maternity care may add challenges to the implementation of PRMs [33]. To fill the gap that maternity care professionals’ views have not been studied widely, this study investigates the local qualified professionals’ attitudes and thoughts towards the implementation of PRMs into maternity care routine.

## Methods

### Research contexts

Finland provides mothers with free maternity care as part of the national health care and social welfare system [37]. The aim is to have equal and safe care available for all women during the course of pregnancy and childbirth [38]. In 2018, 47,913 children were born in Finland [39]. Over 99.5% of all pregnant mothers in Finland seek care from public maternity service [38, 40]. Maternity care in Finland, funded by municipal and state tax revenues, is a mutual effort of authorities and multiple health care professionals and providers [38]. The Ministry of Social Affairs and Health is responsible for guiding the service provision and development. Municipalities or local authorities are responsible for basic services, providing community-based maternity and child health clinic (Neuvola) services as part of primary health care. Neuvola services, led by public health nurses (PHN) and medical doctors, include regular perinatal monitoring, health examinations at predetermined times, health counselling, and family support with attention to relationships and parenting. Hospital districts (20 in total) organize specialized medical care, offer screening and laboratory tests, and run delivery units in their hospitals. Midwives, working at maternity units of hospitals, are the main service providers for childbirth. National Institute for Health and Welfare of Finland (THL) organizes and controls birth registers and follows maternity-related statistics.

**Fig. 1:**
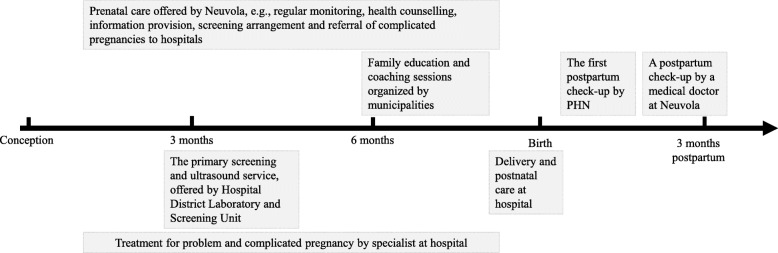
The general maternity care process in Finland

Figure 1 shows the general process of Finnish maternity care. The process begins when a pregnant woman contacts Neuvola and schedules the first appointment. Before or at the first appointment, essential family and health-related information is collected from the pregnant woman and her partner. The pregnant woman is recommended to meet her PHN minimum of eight times, and additionally a medical doctor at least once before birth. The PHN informs the pregnant woman about the availability of the free and voluntary prenatal screening and testing program with different alternatives, and schedules the screening appointments at hospital district screening units. In case of pregnancy complications, Neuvola refers mothers to antenatal clinics or emergency departments of the secondary care hospitals. Upon entering the third trimester, first-time parents can take part in family education and coaching sessions organized by municipalities. When the delivery begins, the mother calls an assigned delivery ward at the secondary care hospital. In the delivery room, midwives take care of the labor process, other professionals including obstetricians, anesthetists, and pediatricians participate when needed, and obstetricians oversee the delivery process. After the birth, the mother and the newborn are transferred to a postnatal unit staffed by midwives, registered nurses and obstetricians. After being discharged from the delivery hospital, mother meets the PHN at home or at a Neuvola clinic within a week for a postpartum check-up. In addition, there is an extensive doctor’s checkup at Neuvola between 2 and 4 months after childbirth.

### Semi-structured interview

In order to explore the acceptability and feasibility of implementing PRMs in Finnish maternity care pathway, this qualitative study applied semi-structured interviews among local professionals to capture and collect experiential, in-depth, and diverse viewpoints. Via interviews, we collected professionals’ views on the applicability of PRMs defined in the ICHOM PCB set for the Finnish context and their concerns on the implementation of PRMs measurement. The interview consisted of two parts applying different data collection techniques and analysis methods. In the first part, professionals were asked to evaluate PRMs defined in ICHOM PCB set in terms of importance of each measure, and appropriateness of the time points of measuring and reporting. Professionals gave scores of importance (0-Not important at all; 1-Nice to have; 2-Important) and appropriateness of the time points (0-Not appropriate at all; 1-To some extend; 2-Appropriate) and provided comments for each measure. In the second part, we explored professionals’ views on the implementation of PRMs in Finnish public maternity services. The concrete interview questions of the second part were developed from *Unified Theory of Acceptance and Use of Technology (UTAUT)* model [41], which was tailored to this project with five constructs, including:

- *Performance* —whether an individual believes /expects that using the system will help to attain gains (i.e. expected usefulness)
- *Effort* —whether an individual believes /expects that there is any ease/complexity and cost associated with the use of the system
- *Conditions* — whether an individual believes /expects that there is any condition, e.g. organizational culture and technical infrastructure, existing to support/hinder the use of the system
- *Perceived risk* -- whether an individual believes /expects that using the system takes a possibility that something unpleasant or unwelcome (harm or damage) will happen to the current system and status
- *Social norms and influence* —whether an individual believes that there is any social norm associated with the use of the system; whether an individual perceives that important others believe he or she should / should not use the system

### Recruitment

Starting from September of 2019, local professionals, including obstetricians, midwives, public nurses, researchers, educators and administrators, who were working in the field of pregnancy and childbirth were approached by two interviewers. Purposive sampling was used as the technique to recruit participants and get a sample that could represent the population regarding the subject under this study. We assumed a certain degree of homogeneity shared by our targeted group of professionals and experts, and according to Guest, Bunce and Johnson [42] we estimated that 15–20 in-depth interviews could be enough to draw some valid conclusions. In order to ensure adequate representativeness of the sample, we continued to conduct the interviews until we reached data saturation, and the additional data made little changes in analytic patterns and themes [43]. Professionals were informed about the purpose and protocol of the interview. All the informants participating in the interviews gave oral or written informed consent.

### Data processing and analysis

Interviews were tape recorded and transcribed. Three Finnish interviews were translated into English. The interviewer who conducted these three Finnish interviews had proficient command of both Finnish and English. She worked with one researcher (AC) in translating and transcribing the Finnish interview records. They listened to the records together, translated the conversations sentence by sentence from Finnish to English, and wrote down the texts in English. All the transcripts were imported into Atlas.ti qualitative data analysis software (V.8.4) and the results were organized with Microsoft Excel. Multiple methods were used to study participants’ views and gain detailed insights of the phenomena being explored. For part 1 of the interviews, assessing measures defined in ICHOM PCB set, we listed the scores and counted the number of professionals for each score. We read all comments given by professionals on each measure, put same and similar comments together, and identified representative viewpoints according to the number of professionals who mentioned them. For part 2 of the interviews, exploring professionals’ views on the implementation of PRMs, we adopted a framework approach embedded with thematic analysis [44, 45]. The primary themes were defined by the modified UTAUT model, which were used as a coding framework. Data were deductively coded and grouped into the predefined themes. Within each primary theme, codes were grouped and categorized to explore patterns and inductively develop sub-themes. Two researchers independently coded transcripts, identified the items relevant to each primary theme of the contextualized UTAUT model, and compared the coding to reach consensus on a coding scheme. Then the two researchers discussed, explored the relationship between the codes, reached an agreement on the prominent sub-themes within each pre-defined primary theme. We enhanced the validity of our analysis and findings by organizing discussions in the research team and getting feedback from some of the professionals we interviewed.

## Results

### Basic characteristics of participants

Altogether 20 professionals participated in interviews. Table 1 summarizes the basic characteristics of participants. Our sample covers the most important and relevant professionals from the main organizations across the maternity care network in Finland. Most interviews were conducted in English, and three in Finnish. The majority of the participants were from the capital region, i.e. Helsinki and Uusimaa hospital district (HUS), which is the biggest hospital district, with roughly 30% of all deliveries in Finland [39]. Each interview lasted from 1.5 to 2 h.

**Table 1:** Basic characteristics of participants

Characteristics of participants	Overall (*N* = 20)
**Gender**	
Male	2 (10%)
Female	18 (90%)
**Work experience** (years of working in the area of pregnancy and childbirth)	
Mean	21.2
Max	40
Min	2
**Current occupation** (one interviewee may have more than one positions)	
Gynecologist & Obstetrician	7
Midwife	5
Public Nurses	2
Manager/administrator	9
Educator/trainer	3
Researcher	4
**Organization** (one interviewee may work for more than one organizations)	
Delivery hospital	10
Neuvola	5
Midwifery school	2
THL	4
**Region**	
Capital region	16 (80%)
Other regions	4 (20%)

### Assessment on PRMs defined in ICHOM PCB set

Table 2 summarizes the professionals’ assessments on patient reported background information items (case-mix variables) and PRMs defined in ICHOM PCB set in terms of importance, and the appropriateness of the timing of the questionnaires. Also, key comments mentioned by at least 25% of the participants are reported. Generally speaking, participants felt that patient reported background information and the PROMs and PREMs included in ICHOM PCB set are important for delivering and developing maternity care in Finland. Compared with PROMs, more participants agreed on the importance of PREMs. However, most participants criticized the timing of data collection suggested in the ICHOM PCB set, which was deemed inappropriate for the Finnish maternity care pathway. A majority of participants considered “birth” (T3) as a problematic time point for data collection, mainly because that it is almost impossible for women who are suffering pain and discomfort during labor to answer PRMs questions and that it would be burdensome for medical staff who are already busy with taking care of labor to execute PRMs data collection. According to some participants, for some measures, e.g. mother-infant attachment and breastfeeding success, it is too early and pointless to inquire about at birth. Some mentioned that in Finland the last maternal checkup is at 2-4 months postpartum (slight difference exists between different healthcare regions), thus it would be more valuable to collect PRMs data during 2-4 months postpartum before the last maternal checkup instead of the evaluation at 6 months postpartum (T5) that might require extra service to be arranged for the mother. Participants noticed that most PROMs and PREMs data had not yet been routinely or systematically collected in Finland, while some PROMs and PREMs related questions had been asked from women via surveys or oral communication at Neuvola and delivery hospitals. Participants agreed that even though ICHOM uses internationally validated instruments in the standard sets, the instruments would need to be tailored for the Finnish population and maternity service context. The major adjustments reported by participants included 1) removing the questions on race, ethnicity and nationality, and replacing it with language or birth place instead, 2) adding health behavior related questions, e.g. asking women about their eating, alcohol consumption, and smoking, and e.g. domestic violence, 3) reframing and simplifying the questions on confidence with breastfeeding, 4) removing “water” from the feeding options, since in Finland it is not recommended to give water to infants less than 6 months old, 5) adding “solid food” to feeding options, 6) using Edinburgh Postnatal Depression Scale (EPDS) as the mental health screening tool during pregnancy, 7) identifying the type of professional, service provider, and service context when asking questions on satisfaction, decision-making, and confidence in healthcare providers, 8) asking questions on satisfaction with care instead of satisfaction with the result of care, 9) removing the item “The delivery room was clean and hygienic” from the questions on birth experience, since cleanliness is not a problem in Finland, and clarifying the item “I came through childbirth virtually unscathed”, 10) redesigning the time points of data collection for each measure. In addition, six participants suggested to add a patient reported measure of fear of childbirth (FOC), an issue that has gained a special attention in Finland. According to the professionals we interviewed, the incidence of FOC in Finland has been increasing recently, affecting 6–10% of pregnant women. Some participants also suggested that questions for partners should be included as well, since they play important roles during the pregnancy and in raising the child.

**Table 2:** Assessing patient reported case-mix variables and patient reported measures defined in ICHOM PCB Set (Interviewee No. = 20)

Patient reported measures and other information	Importance (n)	Appropriateness of the time points of measuring and reporting (n)	Summary of the representative comments and views on the measure (The point was mentioned by at least five participants, 25% of all participants)
Patient reported case-mix variables: Demographic Information, Obstetric and Medical History	Important: 15 Nice to have: 4 Not important: 0 *(one participant did not give scores)*	Appropriate: 19 To some extent: 0 Not appropriate at all: 0 *(one participant did not give scores)*	• The importance of items depends on the professional (obstetrician, midwife, nurse, researcher, etc.), situation (routine prenatal check-up vs. emergence of complications, etc.) and what the data is used for (health care operation, benchmarking, clinical decision making etc.). • In Finland, most of the suggested information has been collected by Neuvola via oral communication and a paper form and recorded in the Neuvola record system. The obstetric and medical information have been collected and recorded by hospitals. Most information can be found in the national birth registry. • Not all information needs to be reported by the woman herself, e.g. some obstetric and medical information. It may be too laborious for the patients to answer all the questions. • The time point of collecting “Multiple gestations” information is problematic, since women usually don’t know if they are carrying multiple fetuses until the first ultrasound. • Race and ethnicity cannot be asked in Finland • Education, occupation, and social support information has not been collected and recorded, but they are important or nice to know for recognizing who may need extra support. • Health behavior information, e.g. eating, alcohol and smoking, should be asked.
PROM Health related quality of life [PROMIS Global10]	Important: 17 Nice to have: 2 Not important: 0 *(one participant did not give scores)*	Appropriate: 9 To some extent: 10 Not appropriate at all: 0 *(one participant did not give scores)*	• Some questions have been asked in Neuvola, but they have not been routinely and systematically collected yet. • A question specifically about the relationship with one’s partner and domestic violence could be added. • Postpartum check-up, if it is just after delivery or in a couple of weeks after delivery, is not a good time point to ask these questions. As shortly after delivery, mother’s hormones change and she starts to realize and get used to the life after having the baby. Three months postpartum could be a good time in Finland to measure women’s quality of life after having a baby.
PROM Incontinence [ICIQ--SF] [Wexner]	Important: 14 Nice to have: 5 Not important: 0 *(one participant did not give scores)*	Appropriate: 6 To some extend:13 Not appropriate at all: 0 *(one participant did not give scores)*	• Some descriptions are quite vague, e.g. large amount, little amount, etc. • In Finland, the information has not been routinely or systematically collected yet. At the postpartum checkup, professionals may check, observe and record it, but it is not regularly reported by women. • Incontinence during pregnancy and childbirth is a very rare problem in Finland. It could be used as a targeted measure for those women who have incontinence problems either during pregnancy or after delivery. • Postpartum check-up, the period defined by ICHOM, is a quite early point to ask incontinence related questions. The most important time point is 3-6 months postpartum, when treatment is necessary if women still have incontinence.
PROM Pain with intercourse [PROMIS SFFAC102]	Important: 16 Nice to have: 4 Not important: 0	Appropriate: 7 To some extent:13 Not appropriate at all: 0	• In Finland, the data has not been systematically or routinely collected yet. Intercourse issues are discussed if women bring them up. • It might be a challenging theme for some ethnic minorities or someone concerning privacy • At the first postpartum check-up, the time point defined by ICHOM, women are not usually having intercourse yet. The best time to measure this in Finland is 3 to 4 months after the delivery.
PROM Confidence with role as a mother [ROLECONF]	Important: 18 Nice to have: 1 Not important: 0 *(one participant did not give scores)*	Appropriate: 8 To some extent: 11 Not appropriate at all: 0 *(one participant did not give scores)*	• Mother’s confidence has not been routinely or systematically measured in Finland. • It is too early to measure mother’s confidence at the beginning of pregnancy; It is more logical to ask about it at the end of the pregnancy.
PROM Mother-infant attachment [MIBS]	Important: 18 Nice to have: 1 Not important: 0 *(one participant did not give scores)*	Appropriate: 5 To some extent: 14 Not appropriate at all: 0 *(one participant did not give scores)*	• In Finland, the information has not been routinely or systematically collected yet. • Feelings towards the baby could be very complicated and vary over time. • It is impossible for women to answer the questions during or just after delivery. • A good moment to measure this in Finland is at 2-4 months postpartum
PROM Maternal confidence with breastfeeding [BFINTENT] [BFCONFID] [BSES-SF]	Important: 15 Nice to have: 2 Not important: 0 *(three participants did not give scores)*	Appropriate: 8 To some extent: 9 Not appropriate at all: 0 *(three participants did not give scores)*	• In Finland, Neuvola and delivery hospitals ask women about their intentions and experiences on breastfeeding, but their confidence has not been systematically measured yet. • There are too many questions, and some are complicated, and should be reframed and recorded. • Breastfeeding Self-Efficacy Scale (BSES) has been translated into Finnish. There are a lot of studies using it. • BSES items seem to be detailed questions about the success or experiences with breastfeeding, not about intention or confidence. • It is good to ask the woman the general questions about intention to breastfeed during the third trimester of the pregnancy. • It is too early to ask BSES questions during or just after delivery. One month postpartum and 3-6 months postpartum are important timepoints for measuring mothers’ confidence with breastfeeding.
PROM Success with breastfeeding [BFSUCCESS]	Important: 17 Nice to have: 1 Not important: 0 *(two participants did not give scores)*	Appropriate: 8 To some extent: 10 Not appropriate at all: 0 *(two participants did not give scores)*	• In Finland, a midwife in the hospital observes and makes records about breastfeeding during postnatal care, and a PHN from Neuvola asks about breastfeeding intentions and experience. • Birth is not good moment to answer the questions and it is hard to say if breastfeeding is successful or not, since the hospital stay is short, and at the beginning breastfeeding may require time and several attempts. In theory, 3 months, 4 months or 6 months after delivery are good moments to measure it. • In Finland, water is not given to a baby before the age of 6 months. At four to 6 months postpartum, “solid food” option should be included.
PROM Postpartum depression_Part 1 [PHQ-9]	Important: 17 Nice to have: 1 Not important: 0 *(two participants did not give scores)*	Appropriate: 12 To some extent: 7 Not appropriate at all:0 *(one participant did not give scores)*	• Mental health of women during pregnancy and childbirth has been a very important issue, and depression is one of the most important PROMs. • PHQ-9 is not used in Finland, but EPDS has been widely used as a screening tool. • During pregnancy, it is good to screen in the beginning to get baseline information, but the 3rd trimester is more important and relevant.
PROM Postpartum depression_Part 2 [EPDS]	Important: 18 Nice to have: 0 Not important: 0 *(two participants did not give scores)*	Appropriate: 11 To some extent: 7 Not appropriate at all: 0 *(two participants did not give scores)*	• Neuvola is now regularly collecting relevant information about depression, using EPDS. • EPDS could be used to screen depression during pregnancy, not just postpartum. • In Finland, women are asked about depression at the two to four-month postpartum checkup
PREM Satisfaction with the result of care [CARESAT]	Important: 19 Nice to have: 0 Not important: 0 *(one participant did not give scores)*	Appropriate: 14 To some extent: 6 Not appropriate at all: 0	• A survey about the delivery experience and satisfaction has been routinely conducted in delivery hospitals. • It is tricky to ask about the result of care that is affected by many factors. It is more logical to ask about care process or to ask about care in general, and then to figure out why women are not satisfied and what needs to be improved. • Different care providers e.g. Neuvola and delivery hospitals, or different professionals, e.g. PHNs, midwives, and doctors, should be evaluated separately. • Instead of 6 months postpartum, 2-4 months postpartum is a more suitable time in the Finnish context to collect data and discuss service experiences with women.
PREM Confidence as an active participant in healthcare decisions [HCR]	Important: 20 Nice to have: 0 Not important: 0	Appropriate: 14 To some extent: 6 Not appropriate at all: 0	• Involving patients in decision-making is a highly important topic that needs to be focused on now. • Different care providers, e.g. Neuvola and delivery hospitals, or different professionals, e.g. PHNs, midwives, and doctors, should be evaluated separately, and certain services, e.g. prenatal screening, should be specifically evaluated. • The key question should be, whether women get the information they feel they need. • It has not been systematically measured in Finland • Instead of 6 months postpartum, 2-4 months postpartum is a more suitable time in the Finnish context to collect data and discuss with women about service experiences.
PREM Confidence in healthcare providers [HCR]	Important: 19 Nice to have: 1 Not important: 0	Appropriate: 14 To some extent: 6 Not appropriate at all: 0	• Different providers should be evaluated separately. • It has not been systematically measured in Finland. • Instead of 6 months postpartum, 2-4 months postpartum is a more suitable time in the Finnish context to collect data and discuss service experiences with women.
PREM Birth Experience [BSS_R]	Important: 20 Nice to have: 0 Not important: 0	Appropriate: 10 To some extent: 9 Not appropriate at all: 1	• This is a core issue in maternity care. • In Finland, Visual Analogue Scale (VAS) is used to measure birth experience and satisfaction. Women answer the question during their postnatal ward episode after the delivery • [BSS_R] items should be calibrated to the general conditions in Finland. For example, the item, “The delivery room was clean and hygienic”, is not relevant in Finland, since there are no problems regarding the hygiene of delivery rooms. The item, “I came through childbirth virtually unscathed”, is ambiguous, and it is difficult for women to understand what is meant by it. • The data should be collected before women are discharged from the postnatal ward or within 1 week of the childbirth.

### Viewpoints on implementation of PRMs

Table 3 displays professionals’ expectations and concerns on the implementation of PRMs in Finnish maternity care. They are organized according to the modified UTAUT framework under five primary themes, i.e. *Performance, Effort, Condition, Risk, Social norms and influence*, and grouped into inductively developed sub-themes. For each sub-theme, we counted the number of professionals who mentioned it to evaluate the importance of each sub-theme compared to other. Table 3 also presents sample quotations.

**Table 3:** Professionals’ views on the implementation of PRMs in Finnish maternity care

**Primary theme 1** **Performance** *whether an individual believes /expects that using the system will help to attain gains (expected usefulness)*	
**Sub-themes (*n*** **= the number of professionals mentioning the sub-theme)**	**Sample quotations**
Maternity care system development (11): improving the whole maternity care chain, building an integrated care system and continuity of care, and enabling national and international comparison	*“The whole maternity care chain will get benefits from this, from primary care to specialized care, and it will help to build continuity of care”* (Interviewee No.4, Educator and Researcher)
Information augmentation (11): getting additional valuable, and structured information from patients, and better understanding women and their needs	*“If women report the problems before the appointment, I can give more information they need and spend more time in talking about the issues. And I can prepare better. I can ask right and important questions. I will know my clients better.”* (Interviewee No. 17, PHN)
Improving management (10): getting a real picture of the overall situation, identifying the deficiencies in the service system, prioritizing the key issues to be addressed, and allocating resources better	*“So as a manager, …I can see bottlenecks, rearrange resources, set the goals more realistically and logically, and guide the work to a right direction.”* (Interviewee No.10, Gynecologist, Obstetrician and Manager)
Improvement of clinical practices (8): improving the service practices and quality of care, and increasing patient satisfaction	*“It is a very valuable way to collect the information that you are not able to get from the current birth registers, and to follow the process and monitor the situation before something happens.”* (Interviewee No. 1, Researcher)
**Primary theme 2** **Effort** *whether an individual believes /expects that there is any ease/complexity and cost associated with the use of the system*	
**Sub-themes (*n*** **= the number of professionals mentioning the sub-theme)**	**Sample quotations**
Data collection, processing and management (19): motivating women to routinely answer PRMs questions, developing advanced IT tools and systems, defining appropriate data access, and ensuring data security	*“Women may feel exhausted to answer questions, especially after the baby is born… We need to motive women and kindly remind them to respond to (PRMs) questions across the care pathway.”* (Interviewee No. 4, Educator and Researcher)
Development of measures and instruments (14): developing relevant, women-friendly and family-oriented measures, forming questions in an acceptable way, and avoiding complicated, long questions and lengthy questionnaires	*“We also need to ask partners and care about partners’ feeling… Fear is the issue to be included in the questions. Currently, we are working a lot to reduce women’s fear of childbirth…There are too many questions defined in the standard. Questionnaire should not be long”* (Interviewee No. 8, Midwife and Manager)
Data utilization and translation (9): properly utilizing the data and analysis results, responding to the emerging issues, providing necessary interventions, exploring the relation between PROMs, PREMs and other outcome measures.	*“If the score (answers to PRM questions) shows a bad situation, we need to think of how to give help and what is the next step. We need to know where to prefer the patient. If we don’t prepare next step or tool (for the emerging issues), it is meaningless to collect the data.”* (Interviewee No. 8, Midwife and Manager)
Integration (7): integrating PROMs and PREMs into current information system and into routine service, making it as a part of care, integrating it with on-going surveys	*“A good way to integrate PROMs and PREMs into the daily service is to collect the PROMs and PREMs before each appointment. At the appointment, medical staff can discuss the emerging problems with patients, and give help or suggestions.”* (Interviewee No. 1, Researcher)
Collaboration and coordination (6): establishing collaboration between different professionals and providers, optimizing information sharing	*“It is challenging for hospitals to collaborate with Neuvola and municipalities. Hospitals and Neuvola should know how to collaborate in information collection and sharing.”* (Interviewee No. 3, Researcher)
**Primary theme 3** **Condition** *whether an individual believes /expects that there is any condition,* e.g. *organizational culture and technical infrastructure, existing to support/hinder the use of the system*	
**Sub-themes (*n*** **= the number of professionals mentioning the sub-theme)**	**Sample quotations**
Current service culture and relevant efforts supporting PRMs implementation (13)	*“Our postnatal ward midwives ask women about delivery experiences...It is mainly for the mother to speak out about their experiences. The scores are recorded. There have been some scale tools used to measure the mother’s experiences,* e.g. *VAS (Visual Analogue Scale).”* (Interviewee No. 6, Midwife)
Busy mother (13)	*“It is hard to do the survey in the delivery room. Women only stay 2 h there after delivery, and there is so much to do already, checking the mother and the baby, going to shower, eating, and a lot of paperwork. After delivery, women are busy with the newborn and don’t have enough time to answer too many questions.”* (Interviewee No. 5, Educator)
Busy staff (9)	*“We are already quite struggling with basic things and daily routines here at this moment. We do get lots of feedback on breastfeeding from mothers, complaining about insufficient information.”* (Interviewee No. 7, Midwife)
Current information infrastructure, systems and tools enabling data collection, processing and management (8)	*“We have started to adopt Apotti system (a regionally uniform social and healthcare information system, integrating all health and social care data, and allowing patients to check medical information, report health status and communicate with professionals). The primary healthcare of Vantaa has already implemented this system. Maybe it is a tool to collect PROMs and PREMs data.”* (Interviewee No. 6, Midwife)
Lack of integration between different providers and regions (8)	*“Delivery hospitals and Neuvola use different information system, so how to collect the information, which organizations collect which information, and how the information could be shared and used…all are challenging.”* (Interviewee No. 1, Researcher)
**Primary theme 4** **Perceived risk** *whether an individual perceives that using the system takes a possibility that something unpleasant or unwelcome (harm or damage) will happen to the current system and status*	
**Sub-themes (*n*** **= the number of professionals mentioning the sub-theme)**	**Sample quotations**
Data bias caused by drop-out and being left out (14)	*“If the questionnaires are too long, patients will be tired of answering questions and will refuse to answer questions”* (Interviewee No. 4, Educator and Researcher)
Improperly utilizing the data (4)	*“If we don’t plan next step or tool (to address emerged problems), it is meaningless and wasteful to collect the data.”* (Interviewee No. 8, Midwife and Manager)
Causing burdens on staff (3)	*“Our staff are too busy and burdensome to collect the data and we do lot of paper works now. We have to concentrate on key stuff.”* (Interviewee No. 5, Educator)
**Primary theme 5** **Social norms and influence** *whether an individual believes that there is any social norm associated with the use of the system; whether an individual perceives that important others believe he or she should / should not use the system*	
**Sub-themes (*n*** **= the number of professionals mentioning the sub-theme)**	**Sample quotations**
Healthcare principles and trends (16): patient-centered care, healthcare equality, integrated care and continuity of care, evidence-based medicine, and value-based healthcare, which are driving the application of PRMs	*“Evidence-based healthcare and patient-centered care will drive the use and implementation of PROMs and PREMs here in Finland”* (Interviewee No. 20, Midwife and Trainer)

#### Performance

Over half of our participants mentioned that implementing PRMs would bring advantages to the overall maternity care system by improving the maternity care pathway, building an integrated and continuous care system, and by enabling national and international comparisons. The same number of participants felt that through PRMs providers would get valuable information on the patients’ perspective and deepen their understanding of patients and their health status. Half of the interviewees emphasized the managerial benefits, including getting a real picture of the overall situation, identifying the deficiencies in the service system, prioritizing the key issues to be addressed, and allocating resources better. Many participants mentioned that PRMs approach would improve health service practices and increase patient satisfaction.

#### Effort

There was a consensus among participants that it would not be easy to collect, process and manage PRMs data. Participants stated that effort should be put into motivating women to routinely answer PRMs questions, developing advanced data processing tools and systems, and administering data access and protecting the data. Fourteen participants suggested that it would be important to develop contextually appropriate measures, which should be understandable and easy for women to respond to and invite the partner to express their feelings and views. Another important aspect mentioned by nine participants was that the data should be properly utilized, and the data should be translated into actions. Some participants pointed out that it was necessary to integrate PRMs into current information systems and into current routine practices. A few of participants realized that it was necessary to establish closer collaboration between different professionals, and between different providers, and to optimize information sharing.

#### Condition

Participants identified both facilitators and obstacles for implementing PRMs in Finnish maternity care. Over half of participants agreed that current culture in the maternity care services in Finland and current trends, e.g. focusing on service experiences and promoting women’s active role in the care process, would support implementation of PRMs. Eight participants believed that the new hospital information system, Apotti (a regionally uniform social and healthcare information system, integrating all health and social care data, and allowing patients to check medical information, report health status and communicate with professionals) [46], to be implemented in HUS hospitals as well as in the primary care in the municipalities in the hospital district would make the collection of ePROMs (electronic PROMs) and ePREMs (electronic PREMs) feasible. The commonly mentioned obstacles for the implementation of PRMs included the following. First, the busy and exhausted mothers might not be motived to give answers to excessive and repeated questions during the care pathway. Second, busy staff would view PRMs as extra work and refused to change their daily routines. Third, in the districts without well-established integration between primary care and secondary care, the disconnection and inconsistency between different providers would make the data collection and transferring challenging.

#### Perceived risk

More than half of professionals noted that there was a high risk of data bias caused by women refusing to answer excessive and repeated questions, dropping out from answering the PRM questionnaires during the care pathway, or being left out due to language barriers or other factors. Other risks mentioned by some professionals included not utilizing the collected data properly and causing an extra burden to the staff.

#### Social norms and influence

Our participants could not identify someone, who could influence their decision to use PRMs. However, they acknowledged that PRMs are promoted by current trends in healthcare, including patient centered care, healthcare equality, integrated and continuous care, and value-based healthcare.

## Discussion

### Main contributions

This is the first study from Finland, the first one from Nordic countries to explore the applicability of PROMs and PREMs defined by ICHOM for maternity care. This study adds valuable insights into the value, acceptability, and feasibility of applying PRMs in the routine practices of maternity services. Specifically, this study observes the acceptability of the emerging potential international standard among highly qualified professionals who have not participated in developing the standard. Previous literature considers the implementation of PRMs, but mainly in other specialties and clinical settings. The previous experience on maternity care PRMs in routine clinical practices is limited in geographical scope. Our study is an effort to fill this gap.

With this study, we gain a preliminary but important view of Finnish professionals’ expectations on the development and implementation of PRMs for improving public maternity care. This deepens the understanding about developing locally applicable PRMs used in maternity care pathway and also lays ground for further efforts to study and implement PRMs in maternity care. While this study can serve as an example for other Nordic countries with similar social contexts and maternity service culture to develop and implement PRMs, it can also help to expand the knowledge base of the implementation of PRMs for developing patient-centered and value-based health care, especially addressing the worldwide shortage on the understanding of applying PRMs in maternity care routine practices. The findings in our study also contribute to the work of developing generic guidance on the implementation of PRMs by taking into account concerns raised by maternity care professionals in a cultural context not studied before.

### Interpretation

The findings of the current study indicate that the PCB set that has not been systematically implemented in Finland yet contains PROMs, PREMs and other patient reported information (i.e. case-mix variables), which are important and useful in delivering and developing maternity care. Similar results have been reported earlier from Netherlands [13, 33]. In particular, PREMs which cover issues like patient’s active role, birth experience, and satisfaction [40] were rated by almost all participants as being “important”. This finding is in accordance with the observations made by Laureij et al. [33] from Netherlands [3]. However, the contents of patient reported measures and time points of data collection need to be customized and adapted to local service institutions, routines and culture. For instance, the question on ethnicity was suggested to be removed. This change was made also in the adaptation of ICHOM PCB Standard Set to the local setting in Kenya [34]. In contrast to findings in Laureij et al. [33], according to which the recommended timing of the data collection was appropriate, our study suggests that the time points of data collection should be adapted to fit into the local service pathway and practices. In addition, due to the changes of physical and mental status during pregnancy and childbirth, women’s capability to answer PRMs questions could be affected. This should be considered in the selection of time points of data collection. Particularly, consistent with Depla et al. [13], our study indicates that collecting data during or just after birth is problematic, causing burden on women and staff. Instead of collecting PRMs data at 6 months after the birth, collection sometime between 2-4 months postpartum would be more practical, as this is the time frame for the final maternal checkup offered in Finland. In the pilot implementation of Kenya, the first survey was administered in the third trimester of pregnancy (28th week of pregnancy) and the timeline of data collection ended 6 weeks postpartum instead of 6 months postpartum [34]. While changes are inevitable, it is worth noting that modifying the recommended questions, some of which are internationally validated scales, and adjusting the time flow will make international comparisons weaker and compromise the advantage of using standard PROMs and PREMs for cross-country benchmarking. Trade-offs have to be made in the development and implementation of the PRMs.

The potential value of PRMs for information augmentation, service and care improvement, managerial improvements, and maternity care system development was recognized by Finnish healthcare professionals and researchers. Our interviewees felt that introducing PRMs into maternity care was strongly recommended. They reported that patient-centered care, healthcare equality, integrated and continuous care, evidence-based medicine and value-based healthcare are the main healthcare principles and trends driving the introduction of PRMs. Professionals also recognized that implementation of PRMs would support current efforts to build a patient-centered and value-based service culture in Finland. They also indicated that current advanced IT infrastructure, including the new information system, Apotti, used in the capital region, would help to make ePROMs and ePREMs collection, processing and management possible. Although the need for well-supporting IT tools and systems has also been emphasized by other similar studies [13, 33, 34], the realization of digital data collection is filled with uncertainty. While two Dutch studies [13, 33] shared their experience that women preferred completing questionnaires digitally at home, the Kenyan study [34] found that the smartphone-based survey completion rates were high at 85% for the antenatal care period but dropped to 38% in the postnatal period [34]. Although it has been regarded as an important, and feasible goal, implementing PRMs in the Finnish maternity care has challenges and risks. For a successful implementation, various steps like developing suitable questions, redesigning the service pathway and protocols, motivating patients to respond to PRMs questionnaire at different time points of pathway, and training professionals to routinely use the data and address raised issues were considered important. Based on their implementation experiences, Depla et al. [13] suggested that professionals would know what issues to address based on women’s responses, but they would not always feel responsible to act upon them or be clear about how to discuss and address the raised issues. Our interviewees particularly criticized the fragmented service system, where the main providers, i.e. Neuvola and hospitals, have not been collaborating well enough, and information sharing and transfer has not been effectively organized. But according to most of professionals, the implementation of PRMs would facilitate the process of integration. Delivering integrated and continuous care has been an important goal announced by the Finnish authorities [37].

### Practical implication

This study describes Finnish professionals’ viewpoints on introducing PRMs and adapting ICHOM PCB patient reported measures into Finnish maternity care. It provides important strategic and practical insights for planning and conducting implementation pilot(s) in the country. The key suggestions derived from this study for preparing a future implementation pilot, which could also be considered for implementing PRMs in other similar contexts include

- Developing a minimum set of patient-reported measures and items, which covers the most relevant and important issues and contains generic, context/condition/disease-specific questions, avoids unethical questions (e.g. race and sexual activity for certain groups), involves the partners’ view, and has data collection time points that align with the local service pathway;
- Integrating patient-reported data into current information systems, merging the patient-reported outcomes and experiences data with clinician-reported data and other medical data, and building an intelligent system to analyze the data and visualize the results;
- Establishing favorable collaboration of multiple stakeholders, identifying a coordinating group for daily operations, and defining roles, responsibilities, and data access for each stakeholder;
- Reforming service process and protocol to integrate PRMs data collection and utilization as part of care, setting sessions to review patient reported data and discuss emerging issues with patients, and organizing necessary follow-ups for health issues revealed by PRMs;
- Motivating women and partners to respond to PRMs questions by informing them of the added value of PRMs, and developing user-friendly digital tools;
- Training users of PRMs data, educating front-line workers to respond to the issues raised through PROM questionnaires, and appointing a task force to respond to issues arising from PREMs data

### Limitation and future studies

With a limited number of interviews and a limited geographical coverage, the results of this qualitative study may not be generalizable to all Finnish hospitals or to professionals in other countries. For the next step, we will conduct comprehensive surveys among professionals in different hospital districts to get a more confirmative and generalizable view on the applicability of PRMs in Finnish maternity care. Another limitation of our study is the lack of views from other stakeholders, i.e. women, partners and families, who would play key roles in the implementation of PRMs in maternity care. Further study should involve women, partner and/or other important stakeholders, and compare and converge viewpoints from different stakeholders for identifying critical issues related to PRMs implementation and making an implementation plan with feasible strategies and practices. In addition, context specificity might limit the scope of the findings, as e.g. culture and IT infrastructure can have a large impact on the professionals’ views on the importance and feasibility of PRMs collection and utilization. Future effort should be made to expand the investigation into other social, cultural and/or institutional contexts for adding more contextual insights into the knowledge base of PRMs implementation, identifying the best practices, and drawing context-transferable and generalizable conclusions. Ultimately, all the knowledge, experiences and insights should turn to comprehensive guidelines and successful actions of implementing PRMs in routine practices.

## Conclusion

This study reveals that professionals working for Finnish public maternity care consider the PROMs and PREMs and other patient reported items recommended by ICHOM to be relevant and important for delivering and developing maternity service in Finland, and see the need to introduce PRMs into Finnish public maternity care system with the promise to improve maternity care. But the implementation of PRMs will require a lot of efforts, e.g. developing suitable questions, redesigning service pathway and protocols, and motivating patients considered important.

## Supplementary Information

**Additional file 1.** Content and time points of patient reported information, i.e. patient reported case-mix variables, patient reported outcome measures, and patient reported experience measures, defined in ICHOM PCB set.

## Data Availability

The dataset generated and analysed for this study is not publicly available due to the restrictions claimed in the research permissions and letter to interviewees. Data are however available from the authors upon reasonable request and with permissions of ethical committees of corresponding hospital districts and municipalities. For requesting the access to data and concerning issues related to the data, please contact with the corresponding author.

## Abbreviations

VBHC: Value Based Health Care
PCC: Patient Centered Care
PRMs: Patient Reported Measures
PROMs: Patient Reported Outcome Measures
PREMs: Patient Reported Experience Measures
ICHOM: International Consortium on Health Outcome Measurement
PCB set: An outcomes and quality measurement set for pregnancy and childbirth developed by ICHOM
THL: National Institute for Health and Welfare of Finland
PHN: Public Health Nurses
UTAUT: Unified Theory of Acceptance and Use of Technology
HUS: Helsinki and Uusimaa Hospital District
ePROMs: Electronic Patient Reported Outcome Measures
ePREMs: Electronic Patient Reported Experience Measures
EPDS: Edinburgh Postnatal Depression Scale
FOC: Fear of Childbirth
